# Seroprevalence of hepatitis B surface antigen (HBsAg) in Bo, Sierra Leone, 2012–2013

**DOI:** 10.1186/s13104-018-3218-8

**Published:** 2018-02-08

**Authors:** Rashid Ansumana, Donald F. Dariano, Kathryn H. Jacobsen, Tomasz A. Leski, Joseph M. Lamin, Joseph Lahai, Umaru Bangura, Alfred S. Bockarie, Chris R. Taitt, Chadwick Yasuda, Moses J. Bockarie, David A. Stenger

**Affiliations:** 1 Mercy Hospital Research Laboratory, Kulanda Town, Bo, Sierra Leone; 2 Department of Community Health and Clinical Studies, Njala University, Bo, Sierra Leone; 3 Center for Biomolecular Science & Engineering, U.S. Naval Research Laboratory, Washington, DC USA; 4 Department of Global & Community Health, George Mason University, Fairfax, VA USA; 5 EDCTP, South African Office, Cape Town, South Africa

**Keywords:** Seroprevalence, Hepatitis B, HBsAg, Bo, Sierra Leone

## Abstract

**Objective:**

The aim of this study was to determine the prevalence of hepatitis B surface antigen (HBsAg) among febrile individuals tested at Mercy Hospital Research Laboratory (MHRL) in Bo, Sierra Leone.

**Results:**

A total of 860 febrile individuals ages 5 years and older were tested by MHRL between July 2012 and June 2013 with a Standard Diagnostics Bioline HBsAg rapid diagnostic test. The overall HBsAg prevalence rate was 13.7%, including a rate of 15.5% among males and 12.6% among females. The HBsAg rate did not differ by child or adult age group (p > 0.5). The prevalence rate in Bo was similar to the 11–15% HBsAg prevalence rates reported in the past decade from other studies across West Africa. Scaling up the infant hepatitis B vaccination program in Sierra Leone will be important for reducing the future burden of disease and premature death attributable to chronic viral hepatitis B disease.

## Introduction

Viral hepatitis is a major cause of morbidity and mortality worldwide, with hepatitis B virus (HBV) the agent responsible for the greatest number of viral hepatitis deaths and disability-adjusted life years (DALYs) lost annually [1]. Viral hepatitis has recently gained new recognition as a public health priority globally and in high-burden regions like West Africa [2]. In 2010, the World Health Assembly adopted a resolution calling for more action to prevent and control chronic viral hepatitis infection in countries bearing a high burden from these diseases [3]. In 2012, the World Health Organization (WHO) released a global action plan for viral hepatitis prevention and control [4]. An updated health sector strategy for viral hepatitis was released by WHO in 2016 [5]. Additionally, the Sustainable Development Goals (SDGs) for 2016–2030 include a target for combatting viral hepatitis as part of broader efforts by the United Nations and its partner agencies and organizations to end poverty, promote human and environmental health, and foster peace and prosperity [6]. Progress toward achieving this SDG target will be monitored with an indicator of hepatitis B incidence, the number of new hepatitis B infections per 100,000 people per year [6].

The rates of deaths and DALYs attributable to hepatitis B in West Africa are among the highest found in any world region [1, 7–9]. HBV is spread through parenteral transmission (that is, through contact with blood or other body fluids via needles, open wounds, or other portals of entry) as well as through sexual contact and perinatal (mother-to-child) transmission during delivery. About 90% of infected infants and some older children and adults who contract HBV fail to clear the virus and develop a chronic infection [10]. People with chronic HBV have an elevated risk of cirrhosis, hepatocellular carcinoma (a type of liver cancer), and other liver diseases. These sequelae increase the risk of premature death in adulthood. While a hepatitis B vaccine is available, it has not been adopted for use in all countries and most adults worldwide were born before hepatitis B vaccination was part of any routine childhood vaccination schedules [11].

There is a need for up-to-date epidemiological data about viral hepatitis so that progress toward achieving the SDG target can be monitored. A lack of recent serosurvey data from many low-income countries means that most current estimates of the burden from viral hepatitis in lower-income areas have been generated by mathematical models like the ones from the Global Burden of Disease (GBD) project rather than being based on field data [1]. New serosurvey data will be valuable for understanding the epidemiological profiles of studied populations. They will also improve the accuracy of GBD estimates in the study countries and their neighbors.

There are few recent studies of viral hepatitis from Sierra Leone. Serosurveys conducted in Freetown in the early 2000s reported hepatitis B prevalence rates of 22% among 198 adults and 6% among 302 pregnant women [12, 13]. A study of 66 young children in the 1990s found an 18% prevalence of hepatitis B [14], and a study of 179 pregnant women in 1995–1996 found an 11% prevalence rate [15]. More recently, a small study of 308 patients of Bo Government Hospital found a 21.4% hepatitis B seropositivity rate [16]. These studies suggest a high burden from hepatitis B in Sierra Leone, but that needs to be confirmed with new and more extensive data. The goal of this study was to determine the current prevalence of hepatitis B infection in Bo, Sierra Leone.

## Main text

### Methods

Mercy Hospital (MH) and the Hospital Research Laboratory (MHRL) are located in Bo, Sierra Leone. Between July 2012 and June 2013, MHRL conducted a research study examining the etiology of febrile illnesses among residents of Bo. To be eligible for this study, individuals had to live in Bo, be at least 5 years old, consent to participation (or, for minor children, have a parent or legal guardian provide consent), and have a fever that they reported began no more than 72 h (3 days) before they arrived at MHRL to seek testing. None of the participants had received hepatitis B vaccination. The research protocol was approved by Njala University (Bo, Sierra Leone), George Mason University (Fairfax, Virginia, USA), the U.S. Naval Research Laboratory (Washington, DC, USA), the Liverpool School of Tropical Medicine (Liverpool, UK), and the Sierra Leone Ethics and Scientific Review Committee.

Hepatitis B surface antigen (HBsAg) is the standard test used to diagnose acute or chronic infection with HBV. HBsAg kits from SD Bioline (Standard Diagnostics, Seoul, Republic of Korea) were used for these tests. The SD Bioline HBsAg rapid diagnostic test (RDT) is reported to have a 98% sensitivity and a 99% specificity [17]. Blood samples were collected using a BD Vacutainer Safety-Lok™ blood collection set with a pre-attached holder (Beckton, Dickinson, and Company, Franklin Lakes, New Jersey, USA). The collected blood was aliquoted into BD Vacutainer tubes (purple-top 5 ml or green-top 10 ml vacutainer tubes for blood plasma and 10 ml SST Gold BD vacutainer tubes for blood serum) and 1.5 ml Eppendorf tubes. A total of 100 μl of blood plasma, serum, or whole blood were pipetted into the sample well of the test kits, followed by application of assay diluent. Results were read 20 min later. For quality control, images of all test kits were uploaded to a cloud database via a portable, battery-operated lateral flow assay reader/imager (Deki Reader, Fio Corporation, Toronto, Canada). The final determination of whether each assay was positive, negative, or invalid was based on visual inspection of the uploaded image.

We calculated the proportion of positive test results by sex and age group. We then used Fisher’s exact test to compare prevalence rates by sex and Chi squared tests to compare prevalence rates by age group.

### Results

The overall HBsAg prevalence among the 860 individuals tested was 13.7% (118/860) (Table 1). For males, the overall prevalence was 15.5% (51/330) and for females the overall prevalence was 12.6% (67/530). There were no statistically significant differences by sex (p = 0.24) or among the four age groups (p = 0.99).

**Table 1:** HBsAg test results by age and sex

Age (years)	Males		Females		Total	
	Number tested	Number (%) HBsAg+	Number tested	Number (%) HBsAg+	Number tested	Number (%) HBsAg+
5–14	37	6 (16.2%)	36	4 (11.1%)	73	10 (13.7%)
15–29	99	18 (18.2%)	215	25 (11.6%)	314	43 (13.7%)
30–44	80	10 (12.5%)	106	15 (14.2%)	186	25 (13.4%)
45 and older	114	17 (14.9%)	173	23 (13.3%)	287	40 (13.9%)
Total	330	51 (15.5%)	530	67 (12.6%)	860	118 (13.7%)

### Discussion

Populations with HBsAg seroprevalence rates above 8% are considered to have a high burden of disease from hepatitis B [8]. The 14% HBsAg seroprevalence rate found in this study for 2012–2013 is well above the 8% threshold. The risk of contracting hepatitis B was not correlated with gender, and the prevalence of chronic disease did not vary substantially by age in post-infant populations. The rate in this study from Bo, Sierra Leone, was lower than 21.4% prevalence found in a more recent, but smaller, study from another hospital in Bo [16]. However, the HBsAg rate reported here is similar to most recent studies from other countries in West Africa, where HBsAg rates reported in the past decade range from 11 to 15%, including studies from Burkina Faso (14%) [18], Ghana (13%) [19], Mali (14%) [20], Niger (15%) [21], Nigeria (14%) [22], and Togo (11%) [23]. The similarity of these results to those from Bo reported here supports the validity of this study in addition to confirming that hepatitis B remains a substantial public health burden across West Africa.

The WHO guidelines for prevention and treatment of chronic hepatitis B infection issued in March 2015 promote several strategies for reducing the future burden from hepatitis B, including (1) vaccinating neonates and infants against hepatitis B to prevent vertical transmission as well as provide protection from exposures later in life, (2) preventing mother-to-child transmission of HBV through use of antiviral therapy for pregnant women with chronic infection, and (3) taking steps to prevent the transmission of hepatitis B virus to susceptible individuals who may come into contact with the blood or body fluids of people with chronic HBV infections [24]. WHO recommends that almost all neonates receive a first dose of hepatitis B vaccine within the first 24 h after birth, with follow-up doses later in infancy [25]. Vaccination of newborns is especially important in places with high HBV endemicity levels [25].

Hepatitis B has been part of the national childhood vaccination schedule in Sierra Leone since 2009. About 83% of babies born in 2015 received three doses of the hepatitis B vaccine [26]. Increasing this percentage will be critical for reducing the future burden of HBV in Sierra Leone. When the first dose is administered within a few hours after birth, the vaccine is more than 90% effective at preventing hepatitis B infection in infants whose mothers are HBsAg positive [25]. Given the high percentage of pregnant women in Sierra Leone who are chronic HBV carriers at risk of transmitting the virus to their neonates, having a vaccine rate below 100% means that many infants are still unnecessarily contracting HBV and being placed at risk of developing chronic infection that may cause serious health problems and premature death later in life. Additional preventable hepatitis B disease may be caused by delays in the timing of the first vaccine dose, a challenge not fully captured in the statistics about 3-dose coverage. Antiviral treatment for the prevention of vertical transmission and the treatment of people who are seriously ill from chronic hepatitis B disease is generally not available in Sierra Leone at present. Opportunities to increase maternal access to these medications should be pursued by the Ministry of Health and Sanitation [27]. In the meanwhile, the limited availability of antiviral medications makes aggressive infant vaccination campaigns even more important.

## Limitations

This study had several limitations. First, the inclusion criterion restricted participants to those who had fevers at the time of testing. This may cause the HBsAg prevalence rate to be overestimated. However, chronic HBV infection is unlikely to be a common cause of febrile illness, so we do not expect that the rate in the study population is considerably different than the rate in the general population. Second, we used a single type of test for HBV and the test kits did not distinguish between acute and chronic infections. However, the vast majority of adult infections in Sierra Leone are likely to be chronic infections that were acquired when the individuals were infants. The strengths of the study include the large sample size and the use of a test that has been shown to have high validity in other studies.

The high rate of chronic hepatitis B infection among adults and children in Bo makes it important to scale up the infant hepatitis B vaccination program in order to protect future generations from the burden associated with chronic liver disease.

## Abbreviations

DALY: disability-adjusted life year
GBD: Global Burden of Disease
HBsAg: hepatitis B surface antigen
HBV: hepatitis B virus
MH: Mercy Hospital
MHRL: Mercy Hospital Research Laboratory
RDT: rapid diagnostic test
SD: Standard Diagnostics
SDGs: Sustainable Development Goals
WHO: World Health Organization
